# An Address

**Published:** 1866-03

**Authors:** 


### AN ADDRESS.

BY DR. FIELD.

[Read before the Michigan Dental Association.]

Gentlemen of the Michigan Dental Association:—At our last annual meeting, held at Ypsilanti, it was "Resolved," that it was the duty of every member of this honorable body to write an essay to be read before this association at its next annual meeting on some subject pertaining our to profession. I am sorry to say that this fact had entirely escaped my mind until some three or four days since, when my attention was called to it by seeing the call of the meeting in the "Register." It was a late hour at which to commence the composition of an essay on any subject that would be of interest to such a body of men as I see around me. I felt very much as the five foolish virgins did when the bridegroom came. I was without oil, or rather, if I had any, it was unhandy to get at. But I wished to write *something*, let it be ever so poor, The edict had gone forth; and write, I must. What to write was the next question. Over that I pondered, until my brain was so muddled that I gave up the attempt, but subsequently made another effort; and the subject that I at last selected was "Dental Societies and their Influence," which subject may be a *little* outside of the intended meaning of the resolution heretofore spoken of. A treatise on some dental specialty may have been meant. If so, I must claim your indulgence. Of such we shall, I hope, have many ere the close of our session. Would that I could wield the pen of an Atkinson, a Taft, a McQuillen, a Buckingham. I might then give you some idea of the *benefit* of dental societies; but as I *can not*, I will, in my own quiet way, tell you what *I* think of them. They are the fountains from which we get our nourishment; and he that does not partake of their life-giving waters, is a sloth and a sluggard. Who is there among you that cannot call to mind some poor moldy brother dentist, who, for the last twenty years, say, has been beating backward and forward over the same track, never going a step beyond, never even daring to dream of such a place as the happy land of Canaan; one that, twenty years ago, would always extract an aching denture, and will *always* do it now; never for a moment believing that it might be saved, by proper and judicious treatment; one who, when he hears "a rasping on the scraper at the door," runs his fingers through his hair to make it stand on end, and, as he supposes, give himself a *professional* air, that he may entrap some unwary victim by his sage look, and lead him to believe that he is possessed of more knowledge and ability than is really safe for one mortal to carry, and not explode; one who, did his unsuspecting patient but know his *real* worth, would shun his blighting touch, as would the insect the dazzle of the candle's alluring blaze, did he but know its scorching heat. Mark my word, gentlemen, that man never attends a dental society or association. *He* knows so much that he can learn no more; and from his immense storehouse of knowledge, no others shall fill *their* barn. If a brother practitioner calls upon him, he is asked to take a seat in the *reception* room until our *knowing one* is at leasure to see him. When he does make his appearance, it is with a repellant air, as much as to say, if you think that you are going to get a peep into *my* "Sanctum Sanctorum," you are much mistaken; the effulgence of *my* greatness is more than you can bear. Such a man, gentlemen, you *always* find an anti-association man. Had he made it his duty, and had it been a pleasure to him to always attend the gatherings of his dental brethren, how different would be both his acts and feelings; how much more would he know; and how much better would he feel towards his neighbors; no great discoveries or inventions are ever perfected by one mind. One may guess an idea before unthought of, and know that when put into harness, it may be of great benefit to his fellow man; yet he may not be able to bring forth that idea into any tangible form. Now if he, after fondling his pet for a time, should throw it aside, it may be lost forever. On the contrary, he gives it to the world, a thousand minds receive it as a diamond uncut; and a thousand pairs of hands set busily to work to put it into shape, that it may be of benefit to the world at large, and to their own pockets in particular. Franklin first snatched the lightning from the clouds; but it was of but little use to the world, until bridled by Prof. Morse. And so it is in our profession. Of the thousands of dentists now practicing in the country, no two think or act exactly alike. You may perform a certain operation in a certain way, and I in quite a different one, and both may be successful; and yet, had we given each other the benefit of our own experience, and explained the *modus operandi* by which we gained a desired result, we might have taken from the two and attained a better one. Another good grows out of our dental societies, and that is our becoming better acquainted with one another. We mingle together, in friendly concert, at our "feast of reason and flow of soul;" and we feel that it is good to be there. He of whom we would yesterday have spoken an unkindly word, now commands our respect. We go home to our different places of business with a good opinion of ourselves and every one else. We all feel at least an inch better than before. Nor do the good results of our meetings stop here. They go forth to the world. We have all learned something; and our patients get the benefit of it, and they *appreciate it*, and that, too, gentlemen, in a way that not only makes our hearts feel light, but also adds a little to our *cash* receipts; some, I presume, will take umbrage at the last clause, and say that our *duty* and a love for our chosen calling, should be the sole motives to sustain us. To them I say that if they choose to eat their bread alone, they are welcome to do so; but, as for *me*, *I* want a little *butter*. Love, duty, and a *proper remuneration* for my labor, must go together; and I always feel that when a patient comes to me to have an operation performed, and comes with full confidence in my ability and integrity, and is willing to pay me what my skill and labor are worth, for the time spent, that I can do better for *him* than for one who takes his seat in my chair, with one hand grasping his pocket book, lest I take more from it than some one else might do. Those that attend all the dental meetings that they can, it costs them time, and it costs them money; and who is to receive the benefit of what they learn while there. *Their Patients*, and they are the ones who should foot the bill.

Thus, Mr. President and gentlemen, have I tried, in as few words as possible, to give you some slight idea of my opinions regarding "dental societies and their influence." May all of us often be permitted to stop at these fountains by the way side, in our journey through life, and drink of the waters, there so freely given, that they may strengthen and nourish us on the weary road, and with hands united in one common brotherhood, may we ever be ready and willing to assist each other over the rough and rugged places, our song shall be love, and our motto, "A long pull, a strong pull, and a pull all together."

